# Temporal and spatial *Mycobacterium bovis* prevalence patterns as evidenced in the All Wales Badgers Found Dead (AWBFD) survey of infection 2014–2016

**DOI:** 10.1038/s41598-020-72297-9

**Published:** 2020-09-16

**Authors:** Paul Schroeder, Beverley Hopkins, Jeff Jones, Terry Galloway, Ryan Pike, Simon Rolfe, Glyn Hewinson

**Affiliations:** 1 Wales Bovine TB Epidemiology Team, APHA Wales, Carmarthen, UK; 2 Wales Veterinary Science Centre, Aberystwyth, UK; 3 APHA, Johnstown, Carmarthen, UK; 4 TB Team, Welsh Government, Cardiff, UK; 5 Centre of Excellence for Bovine Tuberculosis, IBERS, Aberystwyth University, Aberystwyth, UK

**Keywords:** Ecology, Diseases, Molecular medicine, Risk factors

## Abstract

In order to better understand the spatial spread of bovine tuberculosis (bTB) in Wales, an All Wales Badgers Found Dead (AWBFD) survey was carried out from 2014–2016. For Wales, as a whole, there was a significant decrease (p < 0.001) in prevalence of bTB in badgers since a similar survey was carried out in 2005–2006, with a drop from 13.3% to 7.3%. The highest prevalence was observed for the High TB Area East (18.6%), which shares its border with England, and differed significantly (p < 0.001) from the High TB Area West (7.4%). The lowest proportion of carcases diagnosed with the disease (0.7%) was in the Low TB Area, followed by the two Intermediate TB Areas of Wales (2.7%). The *M. bovis* isolates from badgers tended to be similar to the genotypes of cattle in the same area, except in the Low TB Area. The direction of any cross species transmission and the drivers for this cannot be determined from this study. The spatial variations described here support the need for regionally adapted surveillance and control measures for bovine tuberculosis in Wales.

## Introduction

Bovine tuberculosis (bTB) is the most pressing animal health issue for Wales today. The control of bTB in the UK, caused by infection with *Mycobacterium bovis*, is complicated by infection in badgers which act as a wildlife reservoir of infection for cattle^1,2^. Between 1998 and 2009 the number of bTB incidents in cattle with TB lesions and/or bacterial culture in Wales doubled every 4.3 years^3^. Between 2012 and 2016 was a steady decline in bTB incidence and in 2016 seven incidents of TB were detected in every 100 herds under surveillance although recurrent incidents have increased from 37 to 46% since 2007^4^. In 2015 5.1% of herd restrictions resulting from a TB incident lasted > 500 days^5^.

A number of policy changes have been introduced since 2010 to enhance bTB control. Annual routine herd testing was introduced as the default throughout Wales in 2010 with further measures to increase TB test sensitivity, expand cattle controls and enhance management of prolonged breakdowns^5^. Policy changes include the withdrawal (rather than suspension) of Officially Tuberculosis Free (OTF) status on epidemiological grounds rather than presence of lesions or positive bacterial culture (2011), the Intensive Action Area (IAA), the area which had the highest TB breakdown density in Wales, was subjected to a ‘combined cattle controls and BCG badger vaccination programme’ (2012–2015) with passive badger surveillance and six monthly TB testing. In 2014, the enhanced management of persistent breakdowns was initiated, entailing enforcement of biosecurity measures and an immediate increase in the use of targeted cattle testing using the interferon-gamma release assay.

The Wales Regionalised TB Eradication Strategy was introduced in December 2017 and combines a new system of geographic division of Wales away from counties to TB Areas, with bespoke regional cattle controls and eradication milestones to better reflect the heterogeneity of the geographical distribution of bTB in Wales. This approach incorporates previous work by the Animal and Plant Health Agency (APHA) on the concept of Spatial Units. TB Areas were designated according to incidence and prevalence of bTB in cattle from 2010 to 2015 into High, Intermediate and Low Spatial Units (each with a unique ID consisting of a county acronym e.g. PE for Pembrokeshire and a number) and are areas roughly standardised on herd numbers. Typically each Spatial Unit contains 200–225 herds, offering resolution between parish and county level (Supplementary Fig S1 Online)^6^.

An important aspect of the bTB control strategy in Wales is to prevent spread of the disease in cattle and this will require a greater understanding of local transmission pathways of *M. bovis* including the role that wildlife plays inthe spread of bTB to cattle. While the role of badgers in the transmission and spread of bTB in the UK has long been subject to debate, their status as wildlife host of *M. bovis* is not contested^2^. The Krebs Randomized Badger Culling Trial (RBCT) remains the key source of empirical information on this issue; the resulting report concluded that in areas where there was high incidence of bTB there was compelling evidence that badgers were a significant source of infection in cattle^1^. Recent evidence suggests that industry led culling of badgers in England was associated with reductions in cattle TB incidence rates after four years but there were variations in effects between cull areas^7^.

The All Wales Badger Found Dead (AWBFD) Survey commenced in 2014. It is the largest passive surveillance programme of bTB in wildlife in Wales to date and followed several previous studies, including the 2015–2016 Intensive Action Area (IAA) Badger Found Dead report^8^ and the 2005–2006 Badger Found Dead survey^3^. This latter survey found that the prevalence of *M. bovis* in badgers varied between 0 and 26 percent and was associated with the incidence of confirmed bTB in cattle herds in the same area. The survey also found that an infected badger was 12.3 times more likely to be within 5 km of a confirmed cattle bTB breakdown than an uninfected badger. Identification and genotyping of *M. bovis* isolates by spacer oligonucleotide typing (spoligotyping) and variable number of tandem repeat (VNTR) typing is routinely performed in Great Britain^9^. In the 2005–2006 survey, the *M. bovis* genotypes isolated from badgers were mostly identical to genotypes from cattle isolates from the same geographical areas. Genotype diversity in animals 0-30 km apart was greater in cattle than in badgers suggesting that the movement of bovines can affect the distribution of *M. bovis* more than the translocation of badgers^3^.

A more recent study of bTB in badgers in England also supports the sampling of found-dead badgers as a means of establishing TB status in wildlife, observing a co-location *of M. bovis* genotypes in both badgers and cattle herds in Cheshire^10^. Here we describe the results of the AWBFD survey undertaken between September 2014 and December 2016. The study was designed to map the distribution of prevalence of *M. bovis* and its genotype in found dead badgers in Wales and to investigate the spatial relationship between bTB in badgers and cattle. It also aimed to determine if there were changes in prevalence compared with the results from the previous found dead badger survey conducted between October 2005 and May 2006^3^.

## Results

### Prevalence estimate

**Figure 1:**
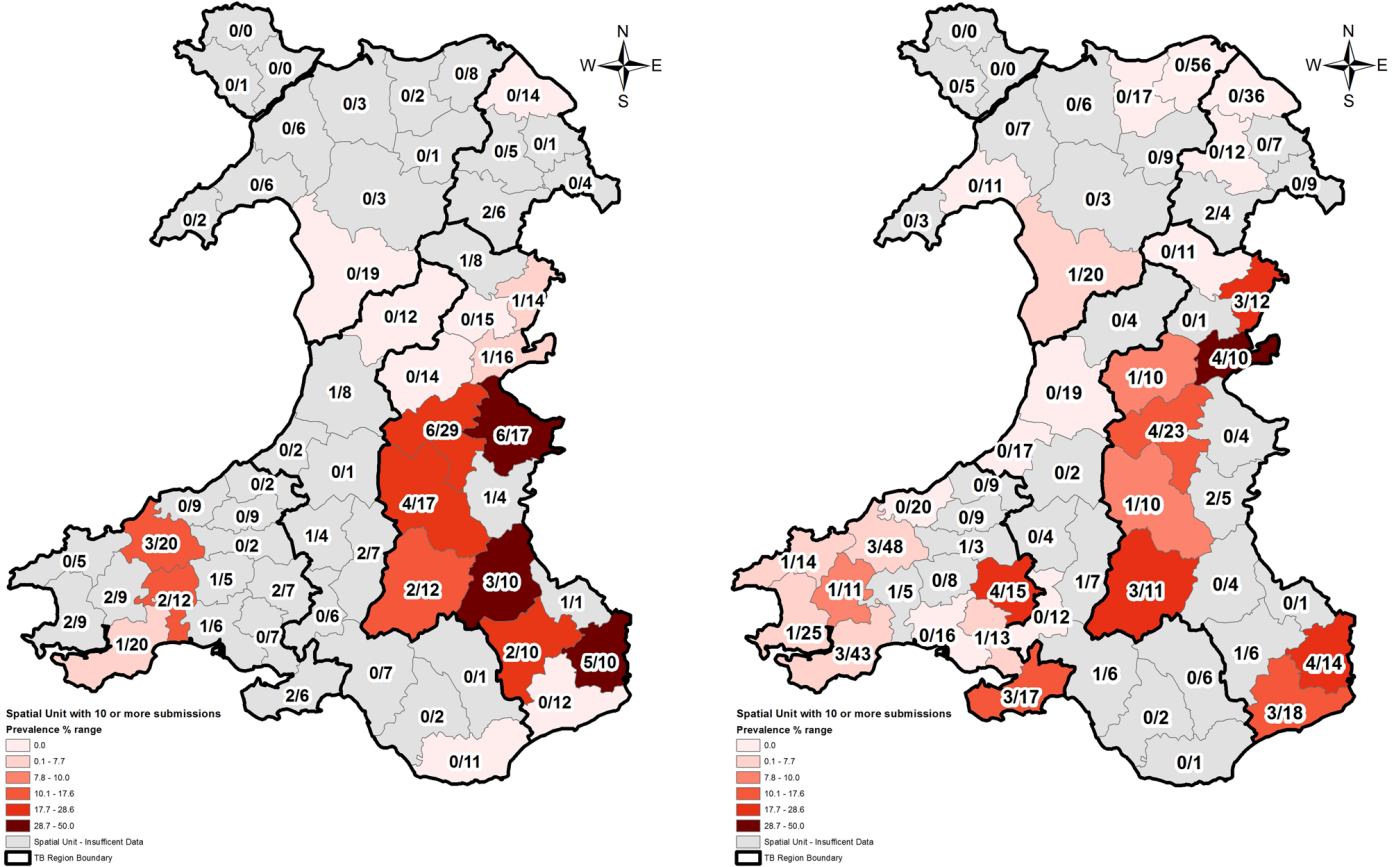
Maps depicting prevalence (% of all submissions that were infected with *M. bovis*) by Spatial Unit in Wales Found Dead badgers (Wales RTA) 2005–2006^3^ and the All Wales Badger Found Dead Survey (AWBFD) 2014–2016. Submissions positive for *M. bovis*/total submissions are given for each Spatial Unit. The colour scale representing prevalence is applied for Spatial Units with at least 10 badger submissions. Mapping software Esri ArcGIS 10.2.2 (https://www.esri.com/en-us/arcgis).

**Figure 2:**
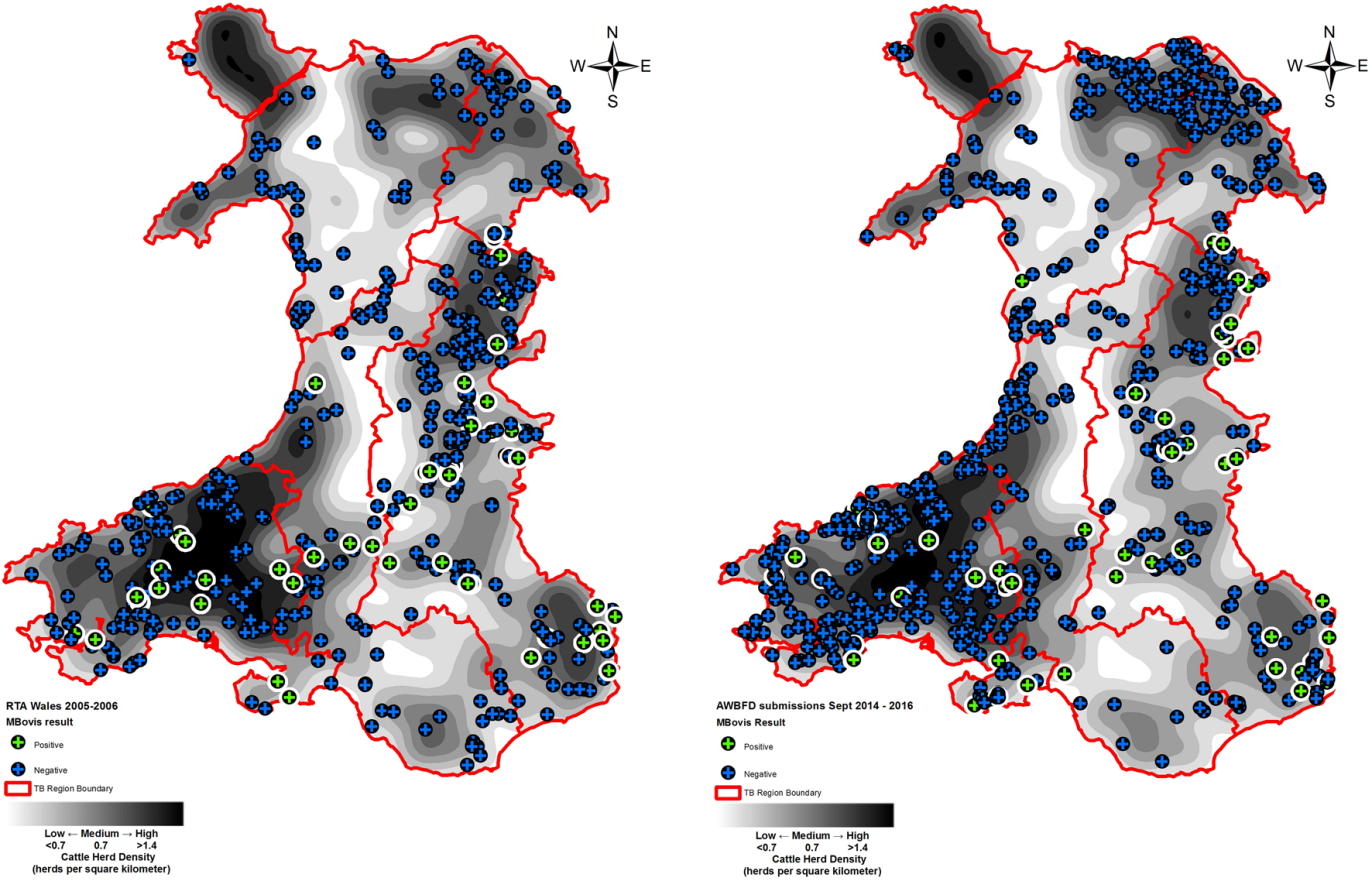
Location and *M. bovis* status of found dead badgers post-mortem examined in Wales in 2005–2006 as part of the RTA survey 2005–2006^3^ and in 2014–2016 as part of the AWBFD. This data is overlaid with Wales TB regionalisation and 2015 herd density data. Herd density info taken from APHA Annual Surveillance Data; mapping software Esri ArcGIS 10.2.2 (https://www.esri.com/en-us/arcgis).

Of the 1863 carcases reported, 841 were collected and 681 (37% of reported carcasses) were submitted for analysis (supplementary table online ST1). *M. bovis* was cultured from 50 carcasses yielding an overall prevalence estimate for Wales of 7% (95% CI 5.6–9.5%). However, significant regional variation was observed (Table 1, Figs. 1, 2). In the High TB Area East the prevalence of infection was 18.6% (12.7–26.2%), compared with 7.4% (4.7–11.2%) for the next highest-prevalence region (High TB Area West). This difference is statistically significant (z = 2.97, p = 0.003). Outside of the two High TB Areas the estimated prevalence for bTB in badgers was low i.e. 2.7% in the Intermediate TB Area (1.1–6.7%) and 0.7% (0.1–4.0%) in the Low TB Area of Wales. Although the differences between prevalence estimates in the High TB Areas and the other areas appear to be compelling, they were only statistically significant (Fisher Exact test) in the case of the Intermediate TB Area and the High TB Area East (p < 0.001), the Low TB Area and the High TB Area East (p < 0.001) and the Low TB Area and the High TB Area West (p < 0.01). The difference between the High TB Area West and the Intermediate TB Area was not statistically significant (p = 0.1).

**Table 1:** Number of submissions and number of badger carcasses found to be positive for *M. bovis* infection by region for Badger Found Dead surveys conducted in 2005–6 (RTA) and 2014–16 (AWBFD); entries for 2005–2006 are based on data reported by Goodchild et al.^3^.

	Submissions		Positives (%)	
	2005–206 RTA	2014–2016 AWBFD	2005–2006 RTA	2014–2016 AWBFD
HE	189	140	33 (17.5)	26 (18.6)
HW *of which IAA*	128 *21*	256 *49*	16 (13.3) *3 (14.3)*	19 (7.4) *3 (6.1)*
IN	30	68	2 (6.7)	2 (2.9)
IM	61	80	4 (6.6)	2 (2.5)
Low	51	137	0 (0)	1 (0.7)
Wales total	459	681	55 (12.0)	50 (7.3)

Abbreviations for the TB Areas in Wales are as follows: High TB Areas East (HE) & West (HW), Intermediate TB Areas North (IN) & Mid/South (IM) and Low TB Area (L). Also included (in italics) are numbers for the Intensive Action Area (IAA). These regions are depicted in Supplementary Fig. S1.

Higher prevalence of infection in males than females was observed; animals with bite wounds were more than twice as likely to be infected with *M. bovis* (17/120 compared to 33/534; z = 3, p, 0.01, Table 2). Females without bite wounds were only half as likely to be infected with *M. bovis* compared to males without bites wounds (11/266 vs 22/268: z = 2.0, p = 0.05).

**Table 2:** *M. bovis* culture results for submitted badger carcasses by sex and presence of bite wounds (n = 654; 27 submissions were not assessed for bite wounds) for the 2014–16 AWBFD survey.

Bite wounds	Female			Male		
	Y (%)	N (%)	Total (%)	Y (%)	N (%)	Total (%)
*M. bovis* + ve	5 (10.9)	11 (4.1)	16 (5.0)	12 (13.2)	22 (7.6)	34 (8.9)
*M. bovis* −ve	41 (89.1)	266 (95.9)	307 (95.0)	79 (86.8)	268 (92.4)	347 (91.1)

Linear regression analysis of annual herd prevalence against badger prevalence estimate for each Spatial Unit with 10 or more badger submissions (Fig. 1) showed that there was a significant correlation for both unadjusted (r = 0.41, p = 0.02) and herd-size adjusted cattle herd prevalence (r = 0.61; p < 0.001, n = 31 for both). For spatial units with 10 or more badger submissions, for those with any positives (regardless of resulting prevalence estimate) the mean annual herd prevalence was 13.9% ± 2.6% (n = 19) compared to Spatial Units with no positives (5.5% ± 2.8%, n = 12, p < 0.001, two sample t test).

In areas where badger vaccination had been carried out, no evidence of adverse reactions associated with vaccination of badgers with Badger BCG was found in any of the badgers examined during this study and BCG was not isolated from any of the samples taken.

### Temporal patterns

The 2005–06 survey yielded a badger prevalence estimate of 13.3% (95% CI 10.4–16.8%) for Wales, based on *M. bovis* cultured from 12% of all submissions and histology typical of bTB in a further 1.3% while the 2014–16 survey gave an estimate of 7.3% (95% CI 5.6–9.5%). The difference is statistically significant (z = − 2.7, p < 0.001), indicating that the observed fall in prevalence of bTB in badgers was not just due to chance alone. There was a 41% relative fall in prevalence estimates between the two surveys in the High TB Area West although this apparent change was not significant (z = 1.3, p = 0.18). In the Intensive Action Area (IAA), which lies within the High TB Area West, there was a pronounced decrease in the badger prevalence estimate with 14.3% (95% CI 2.0–28.0%) in 2005/06 compared with 6.1% (95% CI 0.0–12.0%) in 2014–16, though this was not statistically significant (Fisher Exact Probability test = 0.25) and confidence intervals overlapped. Differences between the prevalence estimates from the 2005–06 and 2014–16 surveys were small for the High TB Area East and the Low TB Area (Table 1). The data also suggest a fall in prevalence in the two Intermediate TB Areas, although Fishers Exact test shows this to be non-significant (p = 0.15).

Over the nine quarters during which badger carcases were submitted there were no clear seasonal prevalence patterns. However, submission numbers varied considerably and there was a recurring trend in that Q1 in 2015 and 2016 represented the two largest samples, or combined, 43% of the submissions total from 22% of the overall sampling period (supplementary Table online ST2). Therefore the results presented may not be an unbiased estimate of the average prevalence during the year. In the previous survey, the authors calculated that the distribution of the months in which the badgers were collected could have caused the mean estimate of the prevalence to be inflated 1.04-fold^3^.

### Comparison of *M. bovis* genotypes in badgers and cattle

**Figure 3:**
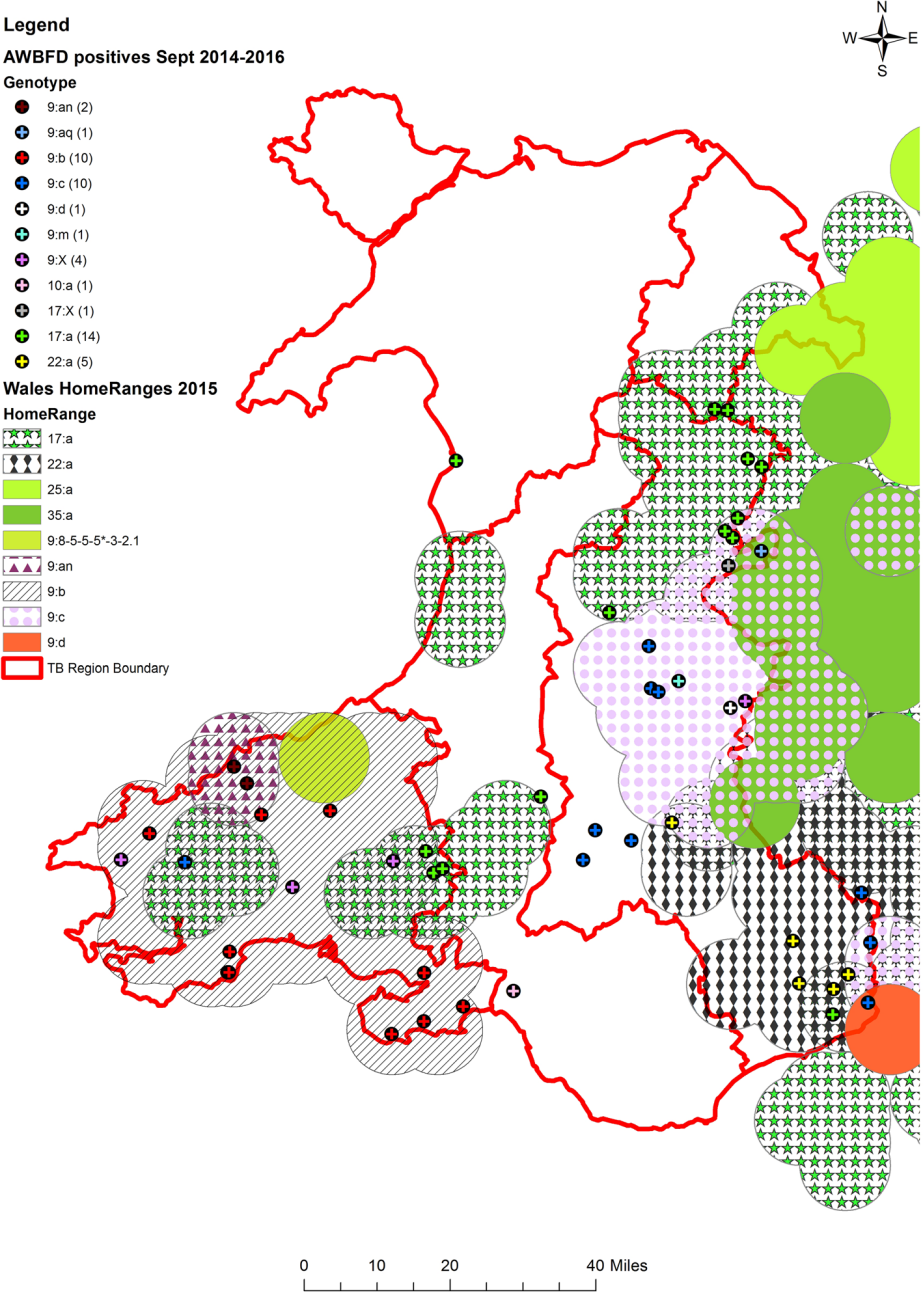
Geographical distribution of genotypes of *M. bovis* isolates from the 2014 to 2016 All Wales Found Dead Badger Survey. Locations and genotypes of *M. bovis* isolates from individual badgers are presented as coloured symbols as per key. The information is overlaid with *M. bovis* genotype ‘home ranges’ derived from *M. bovis* isolates obtained from cattle in 2015 (APHA Annual Surveillance Data) Mapping software Esri ArcGIS 10.2.2 (https://www.esri.com/en-us/arcgis).

Of the 50 *M. bovis* culture positive samples, 45 yielded information at genotype level and five at spoligotype level only (Table 3). Overall nine genotypes were observed and five distinct *M. bovis* genotypes were found at least twice in badgers. These were 9:b (10) 9:c (10), 9:an (2), 17:a (14) and 22:a (5). Spoligotype 9 was the most abundant (30) amongst the *M. bovis* isolates followed by 17: (15) and 22: (5). Most of the genotypes identified in badgers were also found in cattle and tended to have distinct and matched spatial distributions in both animal species i.e. the *M. bovis* genotype in badgers mostly occurred in the ‘home range’ of the locally predominant strain of *M. bovis* in cattle. The 341 M*. bovis* isolates genotyped from cattle in Wales during the 27-month All Wales Badger Found Dead Survey are shown by TB Area (Table 4) and the geographic home ranges are mapped in Fig. 3. Based on the distribution of badger and cattle TB genotype shown in Tables 3 and 4, for three of the five TB Areas (High TB Area East, Intermediate TB Area North , Intermediate TB Area Mid) the Spearman rank correlation coefficient indicated a significant geographic association between badger and cattle genotypes (ρ = 0.83, p = 0.04); while for High TB Area West (ρ = 0.54, p = 0.2) and particularly the Low TB Area (ρ = 0.09, p = 0.8) this was not the case.

**Table 3:** Frequency of genotypes of *M. bovis* in found dead badgers by Wales TB Area for the AWBFD survey between September 2014 and December 2016.

	9:b	9:c	9:an	17:a	22:a	Other	sp.o	Total
HE		10		6	5	3	2	26
HW	10		2	4			3	19
IN				2				2
IM				1		1		2
L				1				1
Total	10	10	2	14	5	4	5	50

Abbreviations for the TB Areas in Wales are as follows: High TB Area East (HE) & West (HW), Intermediate TB Areas North (IN) & Mid/South (IM) and Low TB Area (L); sp.o = spoligotype only.

**Table 4:** Frequency of genotypes of *M. bovis* in cattle by Wales TB Area between September 2014 and December 2016. Abbreviations for the TB Areas in Wales are as follows: High TB Area East (HE) & West (HW), Intermediate TB Areas North (IN) & Mid/South (IM) and Low TB Area (L).

	9:b	9:c	9:an	17:a	22:a	Other	Total
HE	2	46		67	31	16	162
HW	114	1	2	17		11	145
IN	1			19		4	24
IM	4	2		19		1	26
L	2			1	1	4	8
Total	123	49	2	114	32	36	356

Two *M. bovis* isolates from badgers with out-of-home-range genotypes were detected. One isolate (Neath, West Glamorgan, Intermediate TB Area Mid and South) had a genotype with a home range outside of Wales (10:a). This genotype is most frequently found in Oxfordshire, Gloucestershire and Warwickshire^11^. There were no cattle breakdowns with this genotype in the immediate vicinity although three TB breakdowns with 10:a isolated were reported from Glamorgan between 2010 and 2013, all within a 30 km radius from the badger location and with purchased aetiology^12^.

A second isolate (Barmouth, Gwynedd, Low TB Area) with 17:a was found in an area where there was no known home range for any bTB genotype (Fig. 3). There were two herds with TB breakdowns in the Barmouth area in 2014 with the same genotype isolated. The only previous cattle incident with a 17:a genotype in this area had occurred in 2005 and at the time this was attributed to purchased disease from North Powys.

## Discussion

**Figure 4:**
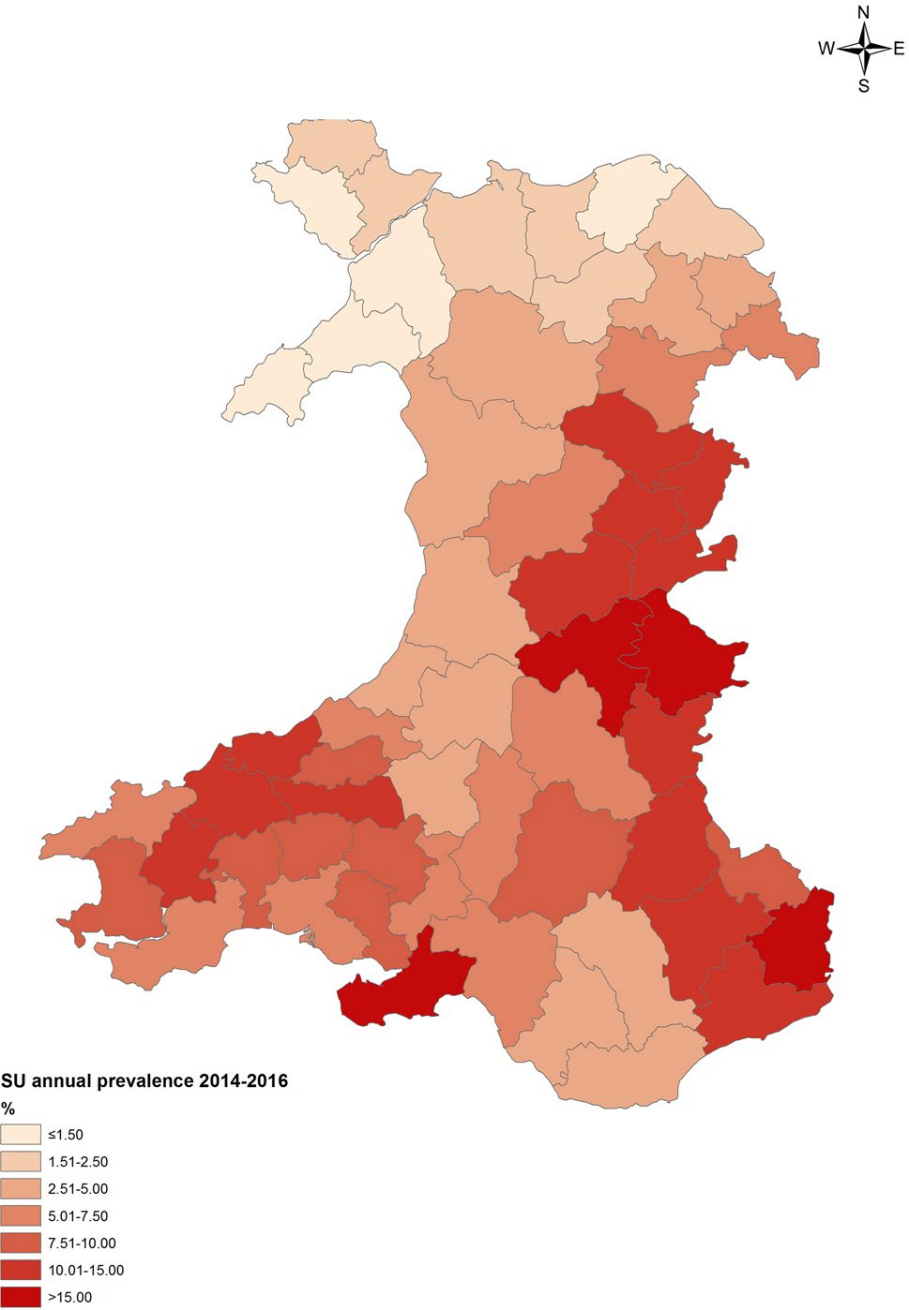
2014–2016 map of annual cattle prevalence (number of herds under bTB restriction at any point of year/registered live herds in region) by Spatial Unit (SU), after direct standardisation to account for variation in herd size. APHA Annual Surveillance Data; mapping software Esri ArcGIS 10.2.2 (https://www.esri.com/en-us/arcgis).

This study has used the collection of road-killed badgers as a means of wildlife surveillance for bTB in Wales. The approach was first reported for Wales in 2012^3^ although such a sampling method had been used successfully in previously reported studies^13–15^. In the present study, 1863 carcasses were reported, 841 collected and 681 carcasses were suitable for further analysis over a period of 27 months between September 2014 and December 2016. *M. bovis* was isolated from 7% of badger carcasses and there was evidence for co-localisation of infection with cattle herd prevalence (Fig. 4) showing a strong association with badger prevalence estimate at Spatial Unit level (Fig. 1) while *M. bovis* genotypes in badgers mostly occurred in the ‘home-range’ of the locally predominant strain of *M. bovis* in cattle (Fig. 3). On a TB Area level, geographical association of *M. bovis* genotypes was statistically significant in the High TB Area East, the Intermediate TB Area North and the Intermediate TB Area Mid Wales. In contrast, the correlation coefficient for the High TB Area West was > 0.5 but non-significant and there was no correlation for the Low TB Area indicating that there is no association between infection status in the two host species in this particular part of Wales, with very low prevalence of bTB in badgers and cattle and all cattle genotypes out of homerange suggesting that infection is brought into this region by the movement of infected cattle. These findings are largely consistent with those from the previous survey in Wales^3^ and a recent investigation in England^10^ which also found an association between the infection status of dead badgers and presence of cattle TB incidents within a 3–5 km radius; also with results from the Randomised Badger Culling Trial (RBCT)^16^ where an association between confirmed herd incidence and badger prevalence at 1 to 2 km was reported. However, as in the previous reports, this study could not determine the direction(s) of transmission between the two species. Recent work^17^ suggests that analysis of whole genome sequence data from badger and cattle isolates in the same geographical locations might allow a better quantification of the roles of badgers and cattle in any inter-species transmission. The authors’ findings suggested that in Woodchester Park, England, within species transmission occurred at higher rates than between species transmission and that transmission occurred more frequently from badgers to cattle than vice versa. As transmission rates may vary from location to location depending on the source of infection and the local epidemiological situation, it is important that those involved in controlling the spread of *M. bovis* understand transmission pathways at a local level in order to better target optimal control interventions and studies involving comparison of whole genome sequences of *M. bovis* isolates from both cattle and badgers present in the same areas might provide a better evidence base with which to do this.

Previous studies have found that the prevalence of infection in badgers was higher in males than females^18–20^ although in most cases these differences were not statistically significant. A recent study in Cheshire^10^ found no significant difference in prevalence by sex or age. In the present study we observed that animals with bite wounds were more than twice as likely to be infected with *M. bovis*. However, females without bite wounds were only half as likely to be infected with *M. bovis* compared to males without bite wounds. This contrasts with the findings from the Cheshire study which reported that no badger carcasses had obvious bite wounds whilst conceding that it was possible that the carcass damage caused by vehicular collision or post-mortem scavengers might have masked their presence^10^. Previous studies have suggested that transmission via biting is possible during aggressive encounters between male badgers^21^ and that badgers with advanced disease are more likely to be bitten due to a loss in social status and competitive ability increasing susceptibility to aggressive behaviour from other badgers in their resident and neighbouring social groups^22^. Such aggressive behaviour by male badgers may be density dependent with the rate of bite wounding increasing with group size and with the number of badgers living in adjacent territories^23^. In our present study there was no system in place to collect badger density data for Wales but the difference in bite wound findings between this study and those from Cheshire^10^ might reflect differences in badger density and associated ranging behaviours of male badgers.

As with the 2005–2006 RTA survey in Wales^3^, there are a number of confounders which may have led to certain sampling bias. As highlighted in previous work^3,10^, such surveys can be influenced by a range of spatial and temporal factors that include: animal density and behaviours, road type and use, seasonality, collection convenience and safety, and engagement by those reporting or collecting carcasses. Larger scale studies would be needed to examine such potential biases. Such studies would need to include effective stakeholder engagement to ensure that similar submission rates are obtained across the whole of Wales.

Although highly specific, the low sensitivity of culture for diagnosing bTB in badgers^24^ is likely to mean the calculated prevalence is underestimated. Up until the 17^th^ December 2012, and therefore at the time of the RTA Badger Found Dead survey, 12 Middlebrook 7H11 agar slopes were used. From 17^th^ December 2012 onwards, the method was amended halving the agar slopes as data generated by APHA had shown no significant difference in recovery rates with the reduced number of agar slopes. While it is appropriate to report this change, there are no grounds to believe that the change in protocol has confounded the temporal comparison by lowering detection rate in the 2014–2016 survey.

Across the High TB Areas of Wales the geographical distribution of infected badgers in 2014–2016 was similar to 2005–2006. The geographical distribution of carcases that tested negative for bTB was roughly similar. A disproportionate increase in Denbighshire and Clwyd was probably due to the substantially increased carcass submissions in the present AWBFD survey. Compared to the 2005–2006 RTA survey, the AWBFD survey reported here generated a larger sample in the Low TB Area. By identifying disproportionately more carcasses in very low prevalence areas one ends up with proportionately more negatives for the whole of Wales. This problem is inherent when dealing with low positive sample sizes. However, if there had been the same number of submissions in the lower prevalence area as in 2005–2006 (51 instead of 137) there would still have been a significant fall in prevalence overall (13.3% to 8.7%; z = 2.4, p = 0.018).

Although we observed a 41% relative fall in prevalence estimates of bTB in badgers between the two surveys in the High TB West Area of Wales this was not statistically significant, highlightling potential limitations relating to submission numbers and methodology inherent in such a survey. This area includes within it the Intensive Action Area (IAA) and a coastal border. Established in 2010 as an area where additional controls would be deployed to address all sources of bovine TB, in both domestic and wild animal species, the IAA has an area of 288 km^2^, most of which in north Pembrokeshire. At the time of its conception the IAA had the highest bTB incidence and prevalence in cattle in Wales. The further controls comprised additional cattle surveillance, enhanced biosecurity measures, additional surveillance for goats and camelids and a programme for badger vaccination (2012–2015) where approximately 70–85% of all badgers in the area would have received the vaccine at least once by the end of the campaign. In 2010 the IAA had 88 TB herd restrictions in 325 herds (27.1%), compared with 38 restrictions in 269 herds (14.1%) at the end of March 2019, which represents a 48% decrease in herd prevalence. This contrasts with figures from the IAA Comparison Area which showed a fall from 142 open cases in 1,250 herds (11.4%) to 97 in 1,130 herds (8.6%) in the same time period, constituting a decrease of 23%.

Thus the additional controls in the IAA aimed at tackling the transmission of *M. bovis* both within and between species appear to have effected a sustained decrease in bTB prevalence in cattle. These encouraging trends suggest that over time, the range of measures applied within the IAA have had a positive impact on disease trends in cattle^25^. The drop in prevalence of bTB in badgers in the IAA from 14.3% in 2005–06 to 6.1% in 2014–16 was not statistically significant and confidence intervals overlapped. The statistical power for this analysis was reduced due to the small sample size and low overall number of positives highlighting that a more systematic study of bTB in bagers in the IAA would have been required to determine whether the measures also had the effect of reducing the prevalence of *M. bovis* in badgers.

In conclusion, this study demonstrated that badger found dead surveys can be a useful approach to surveillance of bTBin wildlife. Findings from this current survey suggest that there has been a decrease in bTB in badgers in Wales with the greatest reductions in prevalence observed in the High TB West Area of Wales. However spatial variation in the prevalence of the disease in badgers was observed, highlighting the need for regionally adapted surveillance and control measures for bovine tuberculosis in Wales.

## Materials and methods

### Locating and collecting badgers

Members of the public, local authorities and countryside organisations in Wales reported the locations of found dead badgers to the Welsh Government or APHA Field Services who recorded the map reference details. The collection by APHA staff of badger carcases that met pre-defined criteria took place between 1^st^ September 2014 and 31st December 2016. There were instances where the collection of reported carcases was not attempted. Reasons for not attempting collection included safety concerns arising from the specific location of the carcass or the non-availability of staff (or other necessary resource) to undertake the collection (supplementary table online ST1). In some further instances collection was attempted but was unsuccessful because either the reported carcase could not be found or the condition of the carcase made it unsuitable for further investigation (for example viscera were herniated externally through wounds, there was severe myiasis (flystrike), the carcase was distended with gas or it was flattened).

Depending on where they were found, the carcases were delivered to the APHA Veterinary Investigation Centres in Carmarthen and Shrewsbury and to the Wales Veterinary Science Centre in Aberystwyth (from 28/01/2016) where they were stored at between 2 and 8 °C for no more than four days before post-mortem examination.

### Post-mortem examination and sampling

Of the 1863 dead badgers reported, 841 were collected and 681 (37% of reported carcasses) were suitable for post-mortem examination. The prevalence of bTB was calculated for suitable carcasses only. The sampling protocol was adapted from the standard and detailed protocols described in a comparison by Crawshaw and others^24^, so that fewer overall samples were taken than the detailed protocol but that the samples chosen were the ones most likely to yield *M. bovis* if present.

The initial external examination comprised the following: measuring the length from nose tip to tail base (cm), assessing and recording tooth wear, recording the sex of the animal and whether female animals were lactating, andrecording any evidence of vaccination: To temporarily identify vaccinated badgers, the guard hairs of the dorsal back (usually caudal) were trimmed and a coloured marker applied at the same site at the time of vaccination. During the external examination of the badger carcases, to attempt to detect recent vaccination, the skin and hair of the back was visually examined for guard hair trimming and coloured marker. Furthermore the examination recorded any evidence of trap injury, or of illegal interference with the animal and the presence and location of bite wounds.

A detailed examination of the lungs, pericardial sac, liver and kidneys was conducted at post-mortem examination. The lungs were examined by making multiple longitudinal incisions approximately one centimetre apart. At least four slices were made in the liver and three slices in the kidney^24^.

Each lymph node of all suitable badgers was incised at least once and a pool of lymph nodes (pool 1) consisting of the retropharyngeal, bronchial, mediastinal and hepatic lymph nodes (or as many as were detectable) was collected for subsequent bacteriological culture. If any gross internal lesions suggestive of tuberculosis were observed or if bite wounds were detected, the lesioned tissue and/or excised bite wounds were added to a separate container (pool 2). The pooled samples for bacteriological culture were preserved in 15 ml of 1% aqueous cetylpyridinium chloride. Samples were sent to the Animal Plant & Health Agency Laboratory inStarcross, Devon on the day of examination, for next day receipt. After taking samples, two or three incisions were made in the muscles of the anterior thigh of both hind legs to look for any potential adverse reactions to BCG vaccination^24^.

### Culture and molecular typing

On receipt at APHA Starcross, the samples were washed in sterile 0.85% saline solution, homogenized by standard methods, inoculated onto six modified Middlebrook 7H11 agar slopes^26^ and incubated at 37 °C. Pool 1, and Pool 2 (if collected), were cultured separately. The slopes were examined weekly from the end of week 2 for a maximum of 12 weeks. *M. bovis* isolates were harvested when growth was sufficient for genotyping and sent to APHA Weybridge.

Genotyping was performed using spoligotyping^27^ and VNTR typing (Exact Tandem Repeat Loci A to F)^9,28^. Spoligotyping confirmed that the isolates were *M. bovis*. Genotypes of *M. bovis* were labelled according to the current APHA convention, using numbers to represent spoligotypes and lower case letters to represent the VNTR pattern within each spoligotype. The same genotyping methods were applied to the cultures of *M. bovis* from cattle slaughtered as part of the national bTB control programme. The cattle genotype home ranges were determined for 2015 (Data Systems Group, Dept of Epidemiological Sciences, Animal & Plant Health Agency Weybridge); for inclusion a genotype had to have been present for at least three years, on at least two different 5 km × 5 km grid squares, in the last 5 years (with a 10 km buffer applied).

### Disease status in cattle herds

The disease status in cattle herds from the five TB regions of Wales has been calculated for all Spatial Units (for definition please refer to introduction) with at least 10 badger submissions and compared with prevalence estimates for the badger populations in these areas. The metric used to describe disease status in cattle is average annual herd prevalence during most of the AWBFD sampling period (2015–2016, number of herds under restriction at any point of year/registered live herds in region). Herd size is a known risk factor for bovine TB^29^; therefore, direct standardisation was used to adjust for varying herd sizes in the Spatial Units^3,30,31^. Briefly, annual stratum-specific prevalence was calculated for each Spatial Unit across four strata (reduced from six in the cited studies due to the small herd denominator in Wales Spatial Units) of herd size and then applied to the reference population, which comprised the sum of cattle populations across all Spatial Units. The standardised population was then used for herd-level disease measures, resulting in a standardised herd prevalence (Fig. 4).

### Badger prevalence mapping

For the Spatial Unit badger prevalence map each Spatial Unit was labelled with the number of submissions, the number of positive results and the resulting prevalence estimate. When added to ArcMap (Esri ArcGIS 10.2.2) the Spatial Unit layer was symbolised using the prevalence value and six pre-defined range values or classes were applied and colour ramped. Spatial Units with less than 10 AWBFD submissions (“insufficient data”) were not colour ramped. All Spatial Units were labelled with the number of positives/number of submissions.

### Statistical analysis

The prevalence of bTB in badgers was estimated among the sampled badgers as in previous studies^3,8^ with the underlying assumption that the carcasses collected were representative of the overall population.

Analysis was performed to test the null hypotheses (H_o_) that:

- There are no differences in badger bTB prevalence between the five TB Areas in Wales.
- There was no change in overall badger bTB prevalence in Wales between the surveys in 2005–2006 and in 2014–2016.
- There was no change in badger bTB prevalence within the five TB Areas between the surveys in 2005–2006 and in 2014–2016.
- There was no change in badger bTB prevalence between the surveys in 2005–2006 and in 2014–2016 within the Intensive Action Area (IAA), site of a 2012–2015 badger vaccination trial.
- There is no correlation between cattle herd prevalence and estimated bTB prevalence in dead badgers in different geographic regions.
- There is no difference in cattle herd prevalence between areas with infected badgers and those with no evidence of bTB in badgers.

For statistical purposes, for the comparisons of prevalence estimates between the TB Areas and over time, both Intermediate TB Areas were combined. All data were tested for normality with the Kolmogorov–Smirnov test (SPSS Version 21 for Windows). Since the dependent variable in these analyses, prevalence, is a rate and fulfilled the criteria for normality, the z-test for comparisons of population proportions was used to test for the statistical significance of differences between prevalence estimates between the TB Areas and over time. A condition of the z-test is that each sample contains at least 10 observations in each category of the dependent variable and for comparisons between samples with less than 10 submissions in at least one category, Fishers Exact test was used^32^. To explore the correlation between prevalence in badgers and cattle, linear regression analysis was undertaken to calculate Pearson’s coefficient of correlation (SPPS 22 for Windows). Spatial Units with > 10 badger submissions with one or more positives were compared with those which had none, using the two sample t test with unequal variances. In order to compare genotypes between badgers and cattle, as in the previous Wales badger survey^3^, the authors calculated the associations between the frequency distribution of the genotypes in badgers and the frequency distribution in cattle for each TB Area. In order to prevent ties and to account for the small number of positive badger submissions, frequencies were adjusted, by replacing them with their deviations from expected values which were calculated as (TB Area subtotal) × (Wales genotype subtotal)/(Wales grand total). A Spearman rank correlation between the ranks of genotypes in badgers and their ranks in cattle was then calculated for each TB Area.

## Supplementary information

Supplementary Figure 1.

Supplementary Tables.

## Data Availability

The datasets analysed during the current study are not publicly accessible due to constraints regarding Official Sensitive disease data in cattle and wildlife but are available from the corresponding author on reasonable request, with necessary redactions of precise geographic coordinates and personal/business identifiers.
