# Author Correction: Temporal and spatial *Mycobacterium bovis* prevalence patterns as evidenced in the All Wales Badgers Found Dead (AWBFD) survey of infection 2014–2016

**DOI:** 10.1038/s41598-021-88888-z

**Published:** 2021-04-29

**Authors:** Paul Schroeder, Beverley Hopkins, Jeff Jones, Terry Galloway, Ryan Pike, Simon Rolfe, Glyn Hewinson

**Affiliations:** 1 Wales Bovine TB Epidemiology Team, APHA Wales, Carmarthen, UK; 2 Wales Veterinary Science Centre, Aberystwyth, UK; 3 APHA, Johnstown, Carmarthen, UK; 4 TB Team, Welsh Government, Cardiff, UK; 5 Centre of Excellence for Bovine Tuberculosis, IBERS, Aberystwyth University, Aberystwyth, UK

Correction to: *Scientific Reports*, 10.1038/s41598-020-72297-9, published online 16 September 2020.

In the original version of this Article, author Jeff Jones was incorrectly affiliated with ‘Veterinary Laboratories Agency, Carmarthen, UK’. The correct affiliation is listed below.

APHA, Johnstown, Carmarthen, UK.

Furthermore, this Article contained errors in Figure 1, where incorrect layers to produce a revised high resolution map were selected. As a result, the values did not represent the period 2014 to 2016 and did not correspond with the badger data shown in the manuscript.

The original Figure 1 appears below.

**Figure 1:**
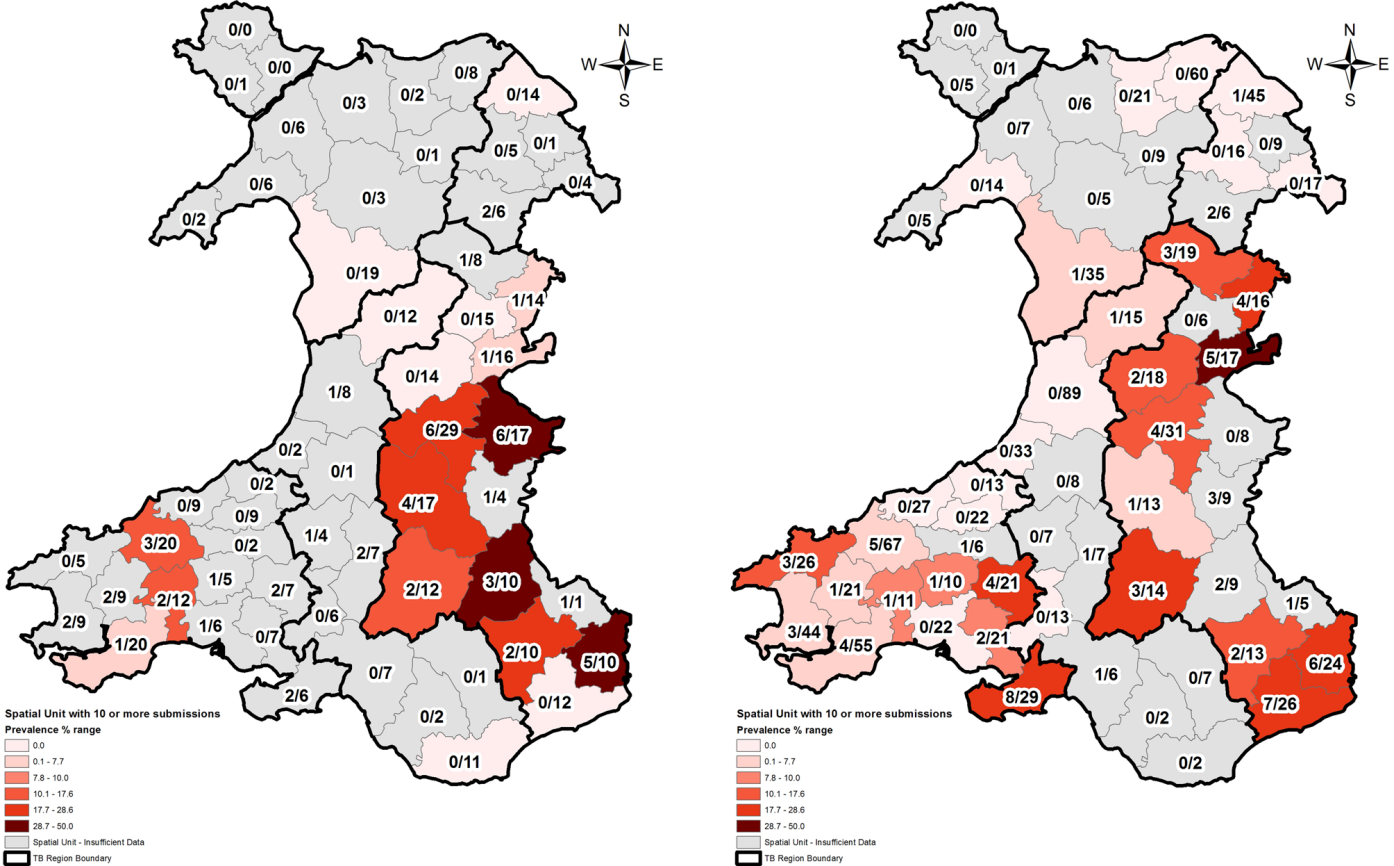
The original incorrect version of the Figure 1.

Additionally, in Table 4, the ‘Total’ column contained incorrect values.

The ‘Total’ reported for the row ‘HE’ changed, where

“26”

now reads:

“162”

The ‘Total’ reported for the row ‘HW’ changed, where

“19”

now reads:

“145”

The ‘Total’ reported for the row ‘IN’ changed, where

“2”

now reads:

“24”

The ‘Total’ reported for the row ‘IM’ changed, where

“2”

now reads:

“26”

The ‘Total’ reported for the row ‘L’ changed, where

“1”

now reads:

“8”

The ‘Total’ reported for the row ‘Total’ changed, where

“50”

now reads:

“356”

The original Table 4 appears below as Table 1.

**Table 1:** The original incorrect version of the Table 4.

	9:b	9:c	9:an	17:a	22:a	Other	Total
HE	2	46		67	31	16	26
HW	114	1	2	17		11	19
IN	1			19		4	2
IM	4	2		19		1	2
L	2			1	1	4	1
Total	123	49	2	114	32	36	50

These errors have now been corrected in the PDF and HTML versions of the Article.

