# Supplementary material for: Temporal and spatial Mycobacterium bovis prevalence patterns as evidenced in the All Wales Badgers Found Dead (AWBFD) survey of infection 2014–2016
Source: Sci Rep. 2020 Sep 16;10:15214. doi: 10.1038/s41598-020-72297-9 (PMC7495426; doi:10.1038/s41598-020-72297-9)
Supplement: Supplementary file 1 — Supplementary Figure 1. [file 41598_2020_72297_MOESM1_ESM.pdf]

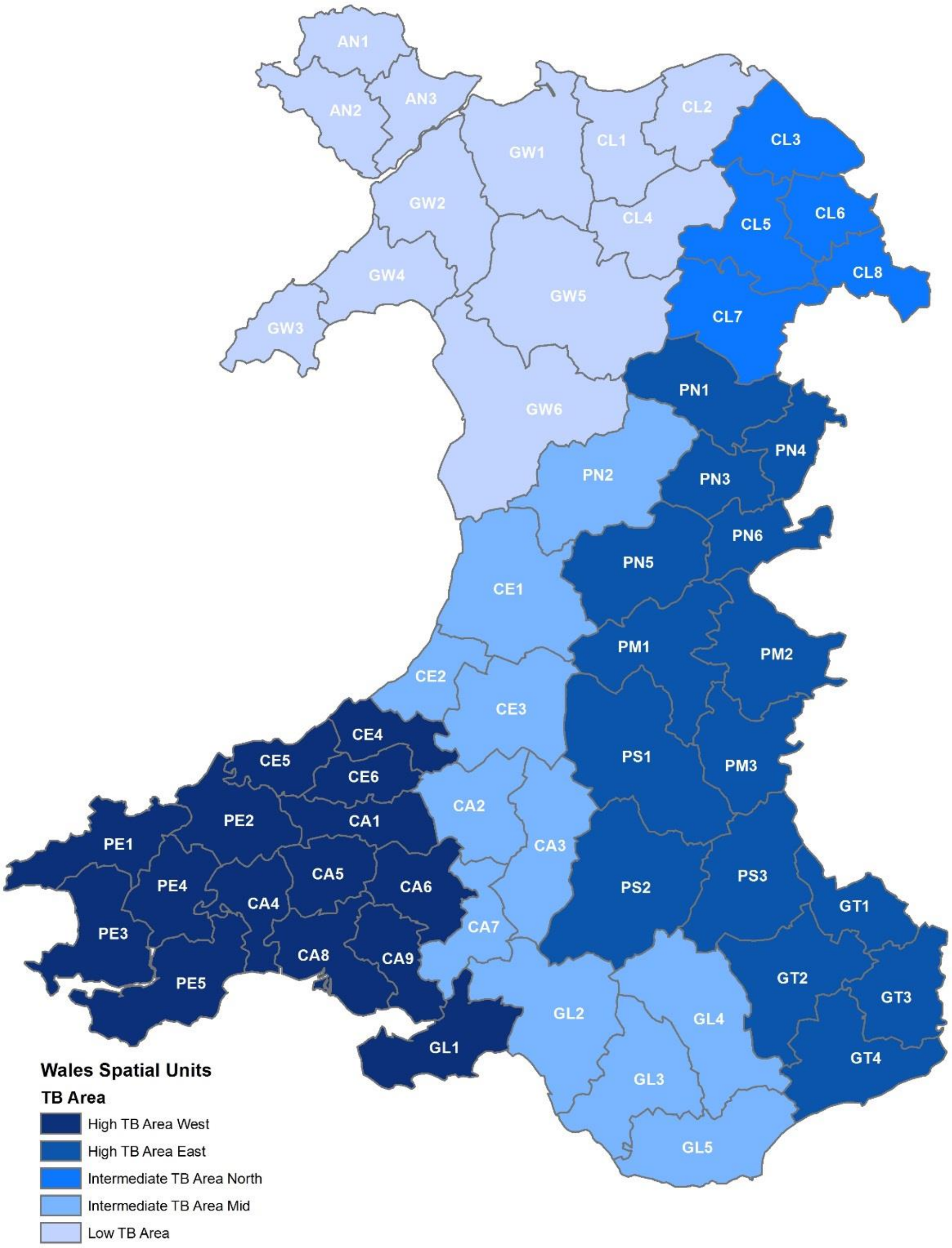

Supplementary Fig S1 – Map depicting Wales TB Regionalisation as of 1/1/2017 with TB Areas & Spatial Units shown. The TB Areas are represented by different shades of blue containing Spatial Units standardised by herd numbers (200-225). Spatial Unit codes indicate previous regional identifiers: AN=Anglesey, CA=Carmathenshire, CE=Ceredigion, CL=Clwyd, GL=Glamorgan, GT=Gwent, GW=Gwynedd, PE=Pembrokeshire, PM=Mid Powys, PN=North Powys, PS=South Powys.

Mapping software Esri ArcGIS 10.2.2 (https://www.esri.com/en-us/arcgis)
