# Supplementary material for: Temporal and spatial Mycobacterium bovis prevalence patterns as evidenced in the All Wales Badgers Found Dead (AWBFD) survey of infection 2014–2016
Source: Sci Rep. 2020 Sep 16;10:15214. doi: 10.1038/s41598-020-72297-9 (PMC7495426; doi:10.1038/s41598-020-72297-9)
Supplement: Supplementary file 2 — Supplementary Tables. [file 41598_2020_72297_MOESM2_ESM.docx]

***Supplementary Table ST1 – Summary of AWBFD 2014-16 survey: reporting and sampling record detailing collection, delivery and submission numbers***

| No of carcass reports to APHA or WG | 1859 |
| --- | --- |
| No of reported carcasses not collected | 1014  142 Dangerous location  48 No resource  275 Not found  504 Not suitable  45 Duplicate reports |
| No of carcases delivered to APHA post-mortem facility | 840 |
| No of submissions for post-mortem examination & culture | 681 |

***Supplementary Table ST2 – Seasonal variation of submissions and badger carcasses found to be positive for M. bovis infection for the High TB Areas East (HE) & West (HW), the Intermediate TB Areas North (IN) & Mid/South (IM) and the Low TB Area (L) as part of the AWBFD survey 2014-16.***

|  | Number of submissions | | | | | Number of positives (%) | | | | |
| --- | --- | --- | --- | --- | --- | --- | --- | --- | --- | --- |
|  | HE | HW | IN | IM | L | HE | HW | IN | IM | L |
| Qtr3 2014 (Sept only) |  | 2 |  |  | 1 |  | (0) |  | (0) |  |
| Qtr4 2014 | 9 | 6 |  | 4 | 1 | (0) | (0) |  | (0) | (0) |
| Qtr1 2015 | 34 | 66 | 16 | 18 | 38 | 9 (26) | 3 (5) | 1 (4) | 1  (6) | 1  (3) |
| Qtr2 2015 | 26 | 36 | 10 | 9 | 18 | 5 (5) | 3 (8) | (0) | (0) | (0) |
| Qtr3 2015 | 19 | 27 | 7 | 3 | 13 | 3 (16) | 2 (7) | (0) | (0) | (0) |
| Qtr4 2015 | 9 | 7 | 9 | 3 | 3 | 2 (22) | (0) | (0) | (0) | (0) |
| Qtr1 2016 | 23 | 35 | 11 | 19 | 28 | 4 (6) | 3 (9) | 1 (9) | 1  (5) | (0) |
| Qtr2 2016 | 9 | 31 | 6 | 9 | 20 | 1 (11) | 2 (6) | (0) | (0) | (0) |
| Qtr3 2016 | 5 | 18 | 3 | 6 | 7 | 1 (20) | 3 (17) | (0) | (0) | (0) |
| Qtr4 2016 | 6 | 29 | 6 | 9 | 8 | 1 (17) | 3 (10) | (0) | (0) | (0) |
